# Axillary masses as clinical manifestations of male sweat gland carcinoma associated with extramammary Paget’s disease and accessory breast carcinoma: two cases report and literature review

**DOI:** 10.1186/s12957-022-02570-w

**Published:** 2022-04-04

**Authors:** Jing Wu, Hongdan Chen, Juanhui Dong, Yong Cao, Wei Li, Fan Zhang, Xiaohua Zeng

**Affiliations:** 1 Chongqing Key Laboratory of Translational Research for Cancer Metastasis and Individualized Treatment, Chongqing University Cancer Hospital, Chongqing, 400030 China; 2 Department of Breast and Thyroid Surgery, Chongqing General Hospital, Chongqing, 401147 China

**Keywords:** Axillary mass, Sweat gland carcinoma, Accessory breast carcinoma, Extramammary Paget’s disease

## Abstract

**Background:**

Male cases of accessory breast carcinoma and sweat gland carcinoma associated with extramammary Paget’s disease of the axilla are uncommon. In clinical diagnosis and treatment, it is necessary to determine the disease carefully and make a reasonable treatment strategy according to the patient’s situation.

**Case presentation:**

We described two male cases of the special tumor with an axillary mass as the first clinical symptom, one of which was diagnosed as accessory breast cancer and the other as sweat gland cancer associated with extramammary Paget’s disease. We treated the two diseases individually in the hopes of providing a reference for the diagnosis and management of diseases with axillary nodules as the initial symptom.

**Conclusions:**

The reports of these two cases can provide reference and corresponding thinking for clinical differentiation of axillary lymphadenopathy caused by different causes and subsequent treatment. These two cases may further enrich the database of rare cases and provide some ideas for the treatment of axillary lymphadenopathy caused by different causes.

## Background

Accessory breast carcinoma (ABC) occurs anywhere along the milk line, with the axilla being the most commonly involved site, followed by the inframammary area [1]. The incidence of ABC is around 0.2–0.6% and that of male ABC is more insidious [2]. Also, sweat gland carcinoma (SGC) is a rare low-grade malignant skin adnexal tumor [3, 4]. Clinically, it mostly occurs in the head and neck skin, followed by the axillary, chest wall, scrotum, and perianal areas with asymptomatic nodular growth. The clinical prognosis for SGC is poor, as it is prone to local recurrence and distant metastasis [5]. Previous studies have demonstrated that the incidence rate of SGC is about 0.05%, which accounts for about 2.2~8.4% of skin malignant tumors. The age of onset is 40–60 years old, and women are more common than men [6, 7]. Moreover, extramammary Paget’s disease (EMPD) is a rare kind of intraepidermal adenocarcinoma involving Paget cells. It is most common in areas with a lot of sweat glands and eccrine glands, like the vulva, genitalia, and perianal region, with a few cases in the armpit as well [8]. Sweat gland carcinoma with EMPD in the axilla is rarer.

A case of male ABC and a case of male SGC associated with EMPD were described in this article. The clinical features and the treatment process of these two patients were very similar. They both started with axillary mass and then underwent local mass resection in a hospital near their home. In the absence of imaging evidence, the final diagnosis mainly depended on the pathology, for example, the shape and size of cancer cells and immunohistochemical characteristics. We hope that by presenting these two cases, we can bring these rare diseases to the attention of medical practitioners and provide some evidence for their diagnosis.

## Case presentation

### Case 1

**Fig. 1:**
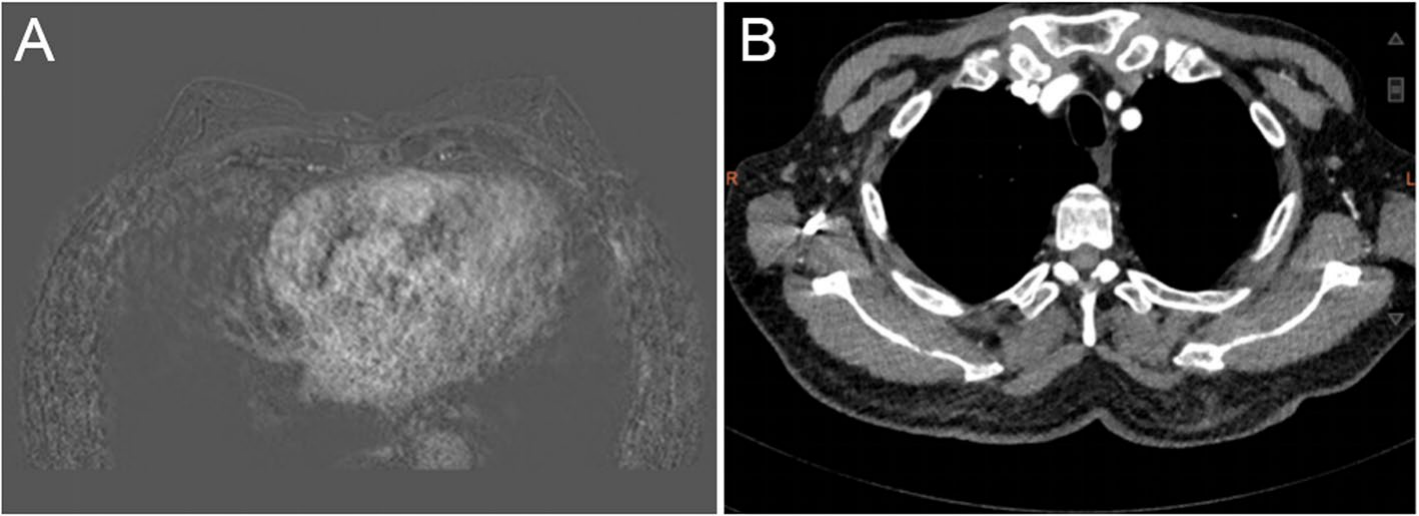
Imaging examination findings of a patient with accessory breast cancer. **A** Breast MRI showed no abnormal mass. **B** CT showed no enlarged lymph nodes in the right armpit

**Fig. 2:**
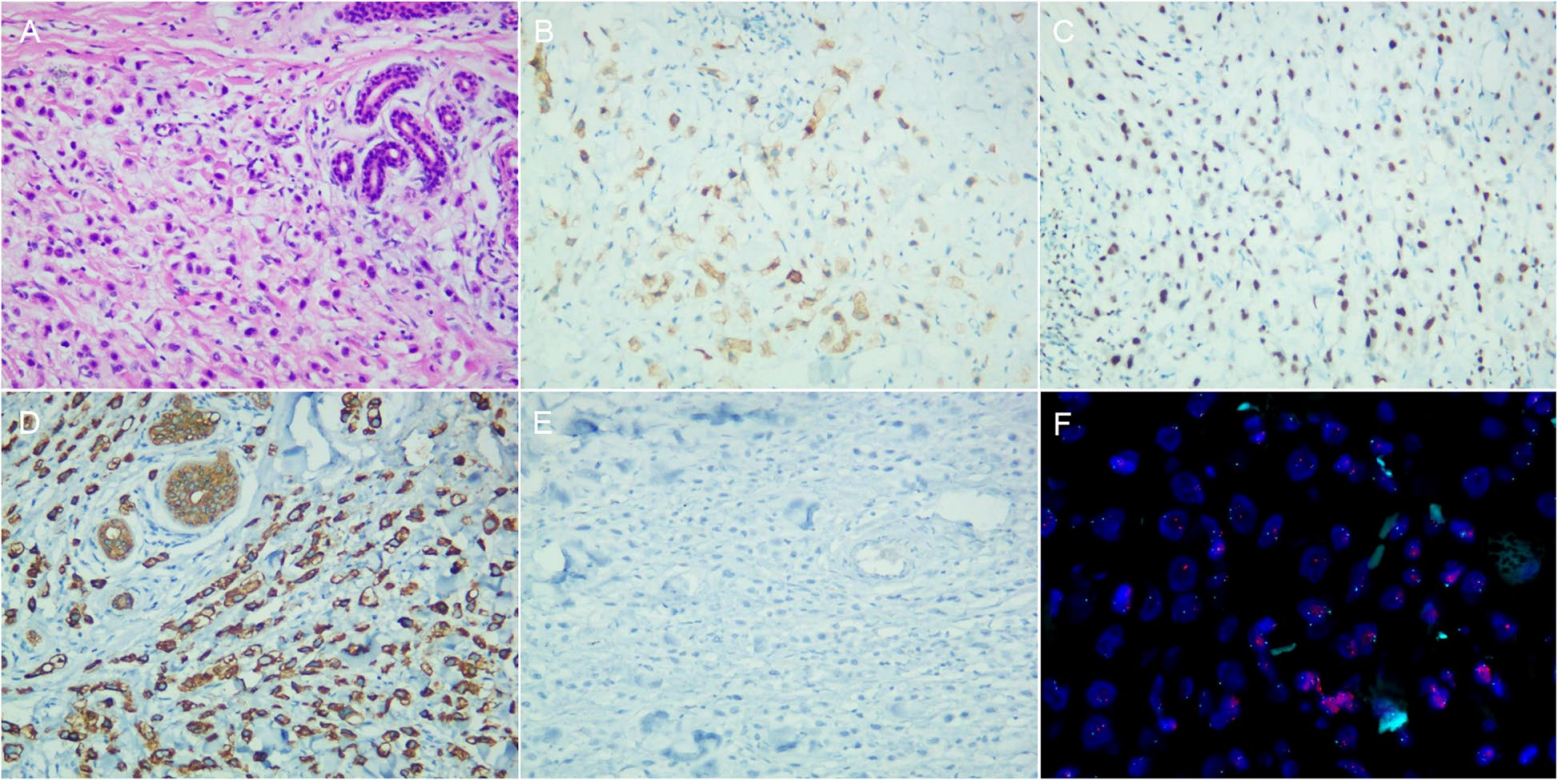
Immunohistochemical staining results of a patient with accessory breast cancer. **A** Photo­micrograph showed poorly differentiated adenocarcinoma (hematoxilin-eosin stain × 20). **B** GCDFP-15 was positive. **C** GATA-3 was positive. **D** CK7 was positive. **E** CK20 was negative. **F** FISH showed no HER2 amplification

An 83-year-old man, a current smoker (180 packs per year) with a history of hypertension and coronary heart disease for more than 10 years, found a mass in his right axilla 1 year ago without redness, swelling, and ache. Until the tumor gradually increased to about 3 cm in size, the patient went to the nearest hospital and underwent local tumor resection on October 26, 2020. Then, he came to our hospital for further treatment. Imaging examinations such as CT, MRI, and PET/CT showed no abnormal enhancement or mass in the breast, no enlarged lymph nodes in the right armpit, and no distant metastasis (Fig. 1). A 3-cm-diameter nodule with skin was found on postoperative pathology. Histologically, poorly differentiated adenocarcinoma was found in subcutaneous fibrous tissue. Positive immunohistochemical staining for anti-gross cystic disease fluid protein-15 (GCDFP-15), GATA-3, cytokeratin (CK), CK7, and EMA and negative for estrogen receptor (ER), progesterone receptor (PR), prostate-specific antigen (PSA), CK20, CDX2, p504S, p63, P40, TTF-1, NapsinA, PLAP, Syn, and CgA. The expression of HER2 was 2+ by immunohistochemistry, and fluorescence in situ hybridization (FISH) showed no amplification, and Ki67 was 20% positive (Fig. 2). After combining the imaging examination, clinical physical examination, and immunohistochemical index, the patient was finally diagnosed with accessory breast cancer.

A daughter of the patient had a history of breast cancer. The doctor advised the patient to complete the BRCA test, but the patient refused. In the evaluation of cardiopulmonary function, no further lymphadenectomy or chemotherapy was performed because the patient was too old and had coronary heart disease and serious arrhythmia. Finally, the patient was treated with intensity-modulated radiation therapy (IMRT) with 6MV X-ray between November 30, 2020, and January 11, 2021. The right operation area, the right axillary lymph node drainage area, and the right accessory breast area were all included in the radiotherapy area. The planning target volume (PTV) dose was 50 Gy/25 F/5 W, and the postoperative gross tumor volume (PGTVtb) dose was 60 Gy/30 F. From surgery to February 2022, the patient’s disease-free survival (DFS) has been more than 15 months.

### Case 2

**Fig. 3:**
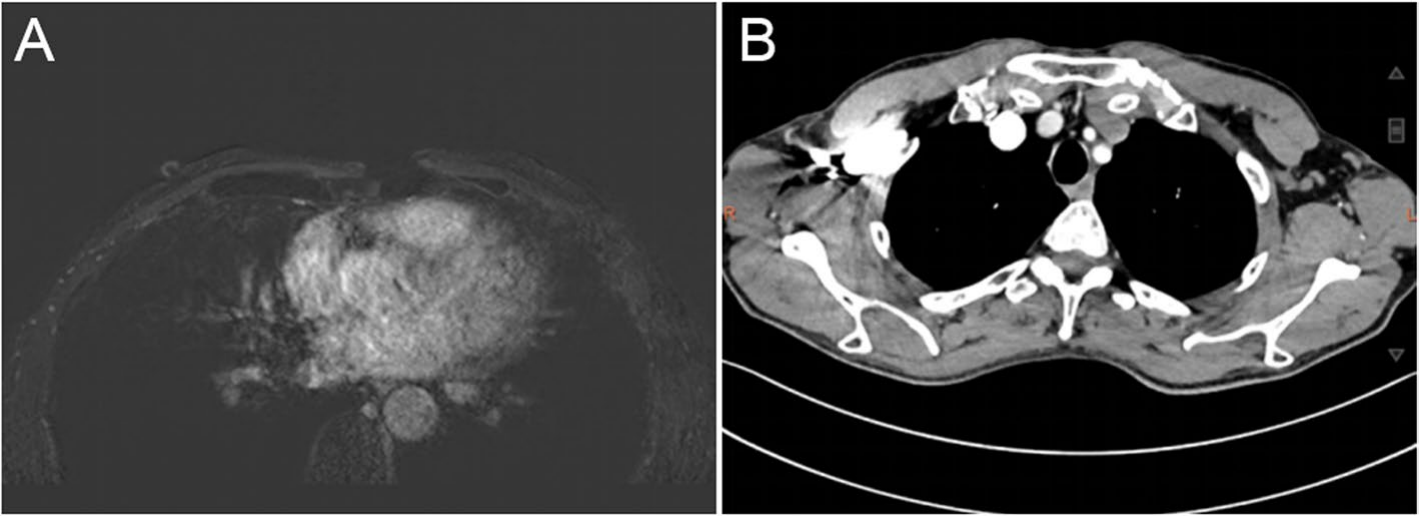
Imaging examination findings of a patient with sweat gland cancer. **A** Breast MRI showed no abnormal mass. **B** CT showed no enlarged lymph nodes in the left armpit

**Fig. 4:**
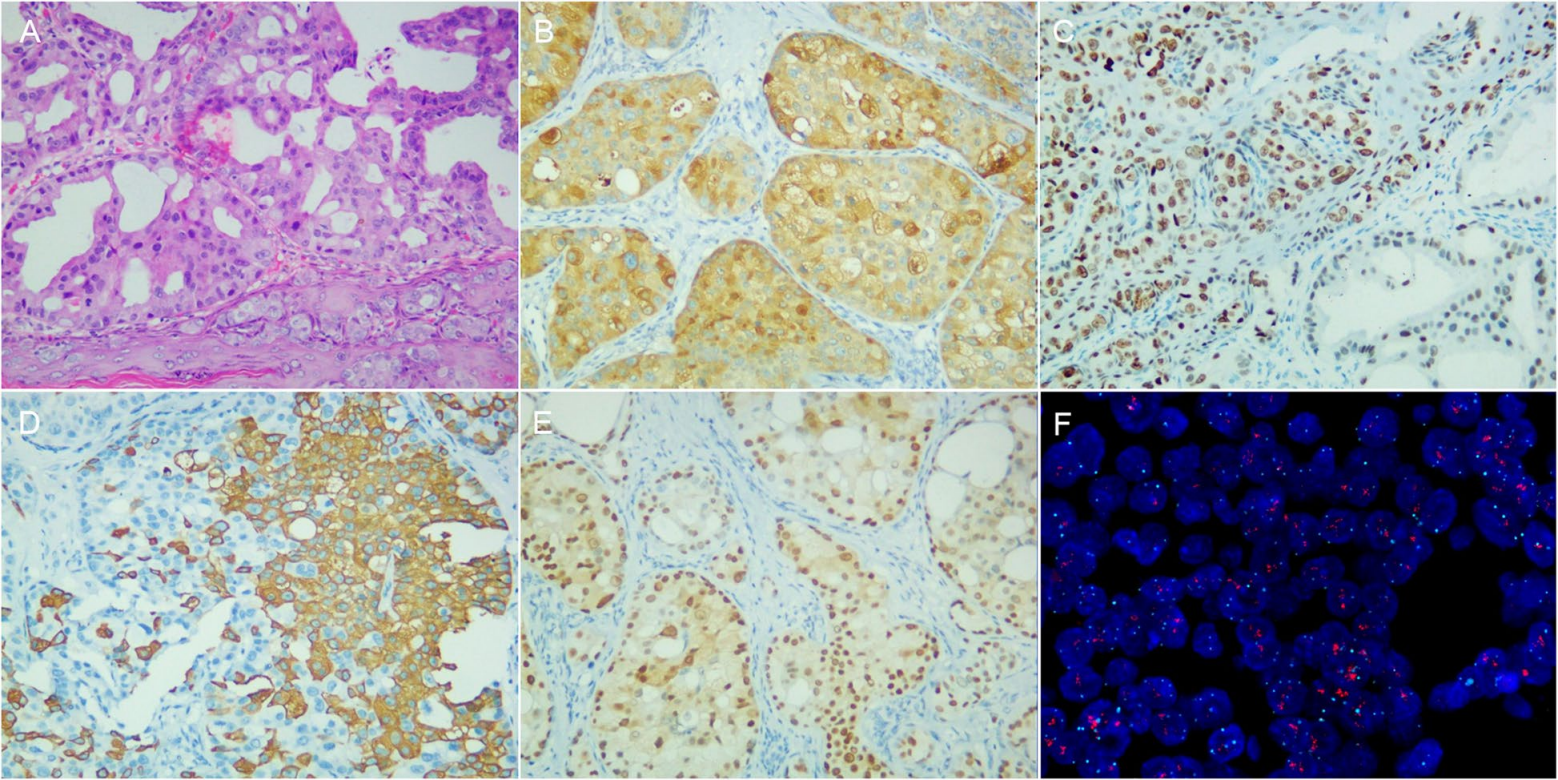
Immunohistochemical staining results of a patient with sweat gland cancer. **A** Histologically showed adenocarcinoma cells coexisting with Paget cells (hematoxilin-eosin stain × 20). **B** GCDFP-15 was positive. **C** GATA-3 was positive. **D** CK20 was partial positive. **E** AR was positive. **F** FISH showed HER2 amplification

A 66-year-old male patient was referred to the nearest clinic for further treatment because he accidentally found a 1.5-cm mass in the left armpit, bulging and red in appearance, without pain and ulceration. On June 20, 2020, after a routine examination, left axillary tumor resection was performed in a nearby hospital. When he came to our hospital, our imaging examinations demonstrated that there was no clear mass in bilateral mammary glands, no obvious enlarged lymph nodes in bilateral armpits, and no metastasis of other organs (Fig. 3). The adenocarcinoma cell, which tends to be sweat gland origin, co-existed with Paget’s cells histologically as the principal component of this malignant tumor. Strong positive immunostaining of GCDFP-15, GATA-3, CK, E-cadherin, and P120-tcn and partial positive immunostaining of CK5/6, CK20, and P53 were observed in sweat gland carcinoma and Paget’s cells, whereas ER, PR, P53, Calponin, MelanA, S-100, HMB-45, and MUC2 did not stain. The expression of HER2 was 2+ by immunohistochemistry, and fluorescence in situ hybridization (FISH) showed amplification, and Ki67 was 20% positive. In addition, androgen receptor (AR) was 40% positive (Fig. 4). The patient was assessed for evidence of sweat gland cancer associated with extramammary Paget’s disease based on these findings.

The patient was previously healthy and had no other complications, and his mother had a history of breast cancer. Thirty days after local resection, the patient underwent extended resection of the left axillary tumor and left axillary lymph node dissection. The pathology after operation displayed a small number of atypical cells under the mucosa of the left axillary tumor resection tissue, and no metastasis was found in the axillary lymph nodes (0/14). From September 4, 2020, to November 13, 2020, four cycles of TC (docetaxel and cyclophosphamide) regimen were used as adjuvant chemotherapy. The patients have been followed up on in good health so far, with no signs of recurrence or metastasis. The DFS was more than 19 months long until February 2022.

## Discussion and conclusions

To our knowledge, ABC in males is extremely rare, and only a few cases are reported at present [9–14]. Accessory breast tissue is a kind of abnormal breast tissue, and its development and pathology are similar to normal breast tissue. Typically, patients, particularly men, are unaware of the presence of accessory mamma until it manifests as inflammation, a benign mass, or a malignant tumor. In total, 60–70% of ectopic breast tumors were occurred by primary accessory breast cancer in the axilla [15–17]. The most common pathological type is invasive ductal carcinoma, just like normal breast cancer [18]. As ABC is rare, early diagnosis is difficult, often resulting in clinical missed diagnosis and misdiagnosis. The differential diagnosis between breast cancer and ABC mainly depends on anatomical location, imaging examination, and pathological diagnosis. In addition, other diseases should also be excluded, such as lymph node metastasis, sweat gland cancer, and lymph node tumor.

The clinical diagnosis of ABC depends on clinical physical examination and imaging examination, such as ultrasound, CT, and MRI. Although some studies suggest that ultrasound and MRI can improve the detection rate of ABC [19, 20], pathology is still the gold standard of diagnosis. The tumor was removed in an outside hospital in our case, and this pathology will play a big role in the final diagnosis. In our case, we made a diagnosis of accessory breast carcinoma of the axilla because there was no evidence of other organ cancer, especially the mammary gland. The majority of the tumors were invasive carcinomas, with a few intraductal components serving as crucial evidence for the diagnosis. The possibility of metastasis from other sites such as gastrointestinal and pulmonary origin also was excluded by different combinations of immunohistochemistry.

Due to the rarity of ABC and the lack of large sample research results, most scholars agree that the treatment of ABC follows the breast cancer guidelines. Some scholars believe that if there is regional lymph node infiltration, radical mastectomy is feasible [21], but Evans et al. [22] found that radical mastectomy or modified radical mastectomy has no obvious advantage in prognosis compared with local resection plus axillary dissection or radiotherapy. Cogswell and Czerny [23] also pointed out that ipsilateral mastectomy had no significant effect on the prognosis of patients with ectopic breast cancer. As a result, local resection of the negative margin combined with axillary lymph node dissection, followed by radiotherapy, chemotherapy, and endocrine therapy, are viable treatment options. The ABC patient in our case was an elderly patient with many underlying diseases, so after comprehensive consideration, we carried out radiotherapy and gave up further lymph node dissection and chemotherapy. Because the patient is triple-negative, there is no need for endocrine therapy and targeted therapy.

SGC is a perplexing area of dermatopathology that was first reported in 1865 by French pathologist V. Cornil [24]. Due to a large number of rare entities, a multiplicity of names to designate the same neoplasms and consequent lack of consensus regarding their classification and nomenclature [25] SGC have traditionally subdivided into four broad groups: eccrine, apocrine, mixed origin (eccrine and apocrine), and other unclassifiable sweat gland tumors [4]. SGC is a heterogeneous tumor with different biological behaviors [26]. It generally presents asymptomatic nodular growth, has local invasiveness, and shows a high recurrence rate. Immunohistochemistry and molecular genetics play a corresponding role in the diagnosis of sweat gland carcinoma after excluding visceral primary adenocarcinoma and skin metastasis [27]. EMPD is characterized by the invasion of the epidermis by Paget cells. The exact pathogenesis of EMPD is not completely clear. The current evidence shows that EMPD is heterogeneous and contains at least two different forms of unique pathogenesis. The primary or cutaneous EMPD forms appear to come from the skin (epidermis or underlying apocrine glands), while the second form is linked to the possibility of adenocarcinoma (in the future) [28]. Its immunohistochemical characteristics are usually positive staining for CK7, CEA, EMA, and GCDFP-15 [29–31]. SGC that co-exists with EMPD is extremely rare. Morgan et al. reported one case of EMPD in the axilla accompanied by underlying apocrine carcinoma, and they also pointed out that 45.5% (5/11) of previously reported axillary EMPDs were associated with an underlying carcinoma [32]. Chiu et al. reported 1 case of unilateral axillary EMPD having underlying adnexal carcinoma [33]. Jung et al. also demonstrated a case of apocrine carcinoma of the axilla associated with EMPD [34]. After carefully excluding lesions in other parts or organs, our case’s final diagnosis was based primarily on pathology, with a large number of adenocarcinoma cells visible on the HE staining section, as well as obvious Paget cells. It is finally classified as SGC coexisting with EMPD when the immunohistochemical characteristics are taken into account..

SGC is easy to recur after the operation, so the first surgical resection and lymph node dissection are the keys to the treatment of the disease. Bogner et al. reported sentinel lymph node biopsy in 5 patients with sweat gland carcinoma before the operation, in which 2 patients had no lymph node metastasis without further surgical treatment. Three patients had lymph node metastasis and then underwent lymph node dissection gradually. In the follow-up of all cases, no local recurrence or distant metastasis was found, demonstrating that a sentinel lymph node biopsy should be performed before the operation to confirm whether there was metastasis and whether additional regional lymph node dissection was required [35]. The treatment of EMPD depends on adequate surgical excision that should be sufficiently wide in surface and depth to eradicate the entire tumor mass. Although the margin of 1 cm is enough, 2 cm is often recommended as a safe margin because the margin of this type of disease is not very clear [36]. The negative margin on frozen sections and Mohs graphic surgery ensures a low recurrence rate [37]. Combined with the characteristics of the above two diseases, our patient finally underwent extended resection and lymphadenectomy. Besides, we also gave the patient systemic chemotherapy (docetaxel and cyclophosphamide) after surgery. We suggested that he receive anti-HER2 targeted therapy, such as trastuzumab, because of his HER2 gene amplification, but the patient ultimately declined due to financial concerns.

In general, the incidence of these two diseases reported by us is very low, especially when both of our cases are male. Although both patients developed an axillary lymph node mass at the same time, their final diagnoses were different. As the two patients had tumor resection in other hospitals, the information about the initial tumor is lacking, and the later diagnosis mainly depends on pathology.

## Data Availability

All data generated or analyzed during this study are included in this published article.

## Abbreviations

ABC: Accessory breast carcinoma
SGC: Sweat gland carcinoma
EMPD: Extramammary Paget’s disease

## References

[1] Teke Z, Kabay B, Akbulut M, Erdem E (2008). Primary infiltrating ductal carcinoma arising in aberrant breast tissue of the axilla: a rare entity. Report of a case. Tumori.

[2] Francone E, Nathan MJ, Murelli F, Bruno MS, Traverso E, Friedman D (2013). Ectopic breast cancer: case report and review of the literature. Aesthet Plast Surg.

[3] Grieco M, Simonacci F, Grignaffini E, Ricci R, Raposio E (2020). Eccrine porocarcinoma: case report and review of the literature. Giornale italiano di dermatologia e venereologia.

[4] Kaseb H, Babiker HM (2021). Eccrine carcinoma. StatPearls.

[5] Brenn T (2020). Do not break a sweat: avoiding pitfalls in the diagnosis of sweat gland tumors. Modern Pathol.

[6] Nair PA, Rathod KM, Chaudhary AH, Pilani AP (2013). Sweat gland adenocarcinoma of scalp. Int J Trichol.

[7] Nizawa T, Oshitari T, Kimoto R, Kajita F, Yotsukura J, Asanagi K (2011). Early-stage mucinous sweat gland adenocarcinoma of eyelid. Clin Ophthalmol (Auckland, NZ).

[8] Lam C, Funaro D (2010). Extramammary Paget’s disease: summary of current knowledge. Dermatol Clin.

[9] Bi L, Li J, Shi Z, Zhu Z, Lu Z (2015). Male accessory breast cancer successfully treated with endocrine therapy: a case report. Oncol Lett.

[10] Bi M, Li D, Su Y, Sun P, Gao Y (2020). Male axillary accessory breast cancer: a case report. Medicine.

[11] Lin Y, Wang Y (2012). Case report of a male primary breast carcinoma of axillary accessory mammary gland. Clin Breast Cancer.

[12] Yoshida Y, Sakakibara A, Watanabe T, Noto K, Sakita K, Sakai Y (2012). Extraordinarily large protruding accessory breast cancer in a man. J Am Acad Dermatol.

[13] Zhong GB, Ye XQ, Liu JL, Xiao SZ, Huang QH, Wei W (2018). Male accessory breast cancer on the abdominal wall: a case report and literature review. OncoTargets Ther.

[14] Takahashi E, Terata K, Nanjo H, Ishiyama K, Hiroshima Y, Yamaguchi A (2021). A male with primary accessory breast carcinoma in an axilla is strongly suspected of having hereditary breast cancer. Int Cancer Confer J.

[15] Amsler E, Sigal-Zafrani B, Marinho E, Aractingi S (2002). Ectopic breast cancer of the axilla. Annales de dermatologie et de venereologie.

[16] Marshall MB, Moynihan JJ, Frost A, Evans SR (1994). Ectopic breast cancer: case report and literature review. Surg Oncol.

[17] Youn HJ, Jung SH (2009). Accessory breast carcinoma. Breast Care (Basel, Switzerland).

[18] Yerra L, Karnad AB, Votaw ML (1997). Primary breast cancer in aberrant breast tissue in the axilla. South Med J.

[19] Nelemans PJ, von Meyenfeldt MF, van Engelshoven JM. The additional diagnostic value of ultrasonography in the diagnosis of breast cancer. Arch Intern Med. 2003;163(10):1194-9. 10.1001/archinte.163.10.1194 12767956

[20] Capobianco G, Spaliviero B, Dessole S, Rocca PC, Cherchi PL, Ambrosini G (2007). Lymph node axillary metastasis from occult contralateral infiltrating lobular carcinoma arising in accessory breast: MRI diagnosis. Breast J.

[21] Tjalma WA, Senten LL (2006). The management of ectopic breast cancer--case report. Eur J Gynaecol Oncol.

[22] Markopoulos C, Kouskos E, Kontzoglou K, Gogas G, Gogas J (2001). Breast cancer in ectopic breast tissue. J Gynaecol Oncol.

[23] Cogswell HD, Czerny EW (1961). Carcinoma of aberrant breast of the axilla. Am Surg.

[24] Gates O, Warren S, Warvi WN (1943). Tumors of sweat glands. Am J Pathol.

[25] Ahn CS, Sangüeza OP (2019). Malignant sweat gland tumors. Hematol Oncol Clin North Am.

[26] Larson K, Babiker HM, Kovoor A, Liau J, Eldersveld J, Elquza E (2018). Oral capecitabine achieves response in metastatic eccrine carcinoma. Case Rep Oncol Med.

[27] van der Horst MPJ, Brenn T (2017). Update on malignant sweat gland tumors. Surg Pathol Clin.

[28] Kanitakis J (2007). Mammary and extramammary Paget’s disease. J Eur Acad Dermatol Venereol.

[29] Liegl B, Leibl S, Gogg-Kamerer M, Tessaro B, Horn LC, Moinfar F (2007). Mammary and extramammary Paget’s disease: an immunohistochemical study of 83 cases. Histopathology.

[30] Shepherd V, Davidson EJ, Davies-Humphreys J (2005). Extramammary Paget’s disease. BJOG.

[31] Yang WJ, Kim DS, Im YJ, Cho KS, Rha KH, Cho NH (2005). Extramammary Paget’s disease of penis and scrotum. Urology.

[32] Morgan JM, Carmichael AJ, Ritchie C (1996). Extramammary Paget’s disease of the axilla with an underlying apocrine carcinoma. Acta Derm Venereol.

[33] Chiu CS, Yang CH, Chen CH (2011). Extramammary Paget’s disease of the unilateral axilla: a review of seven cases in a 20-year experience. Int J Dermatol.

[34] Jung HR, Kwon SY, Son D (2015). Apocrine carcinoma of the axilla associated with extramammary Paget’s disease: a case report and review of the literature. J Pathol Transl Med.

[35] Bogner PN, Fullen DR, Lowe L, Paulino A, Biermann JS, Sondak VK (2003). Lymphatic mapping and sentinel lymph node biopsy in the detection of early metastasis from sweat gland carcinoma. Cancer.

[36] Murata Y, Kumano K (2005). Extramammary Paget’s disease of the genitalia with clinically clear margins can be adequately resected with 1 cm margin. Eur J Dermatol.

[37] Hendi A, Brodland DG, Zitelli JA (2004). Extramammary Paget’s disease: surgical treatment with Mohs micrographic surgery. J Am Acad Dermatol.

